# Acrylic Finger Prosthesis: A Case Report

**DOI:** 10.7759/cureus.30974

**Published:** 2022-11-01

**Authors:** Shreya Colvenkar, Shanthi Priya Kota, Manasa Chalapathi, Gayathri Bandari, D Sriteja

**Affiliations:** 1 Department of Prosthodontics, MNR Dental College and Hospital, Sangareddy, IND; 2 Department of Prosthodontics, S. Nijalingappa Institute of Dental Sciences, Kalaburagi, IND

**Keywords:** hand, amputation, prosthesis, finger, acrylic

## Abstract

Partial-finger amputations not only affect the function of the hand but also the psychology of patients. Amputation can be caused by traumatic injuries, congenital defects, or malformations. Patients will benefit esthetically as well as functionally when a finger prosthesis is uniquely sculpted for each patient. This article describes the fabrication of a custom-made finger prosthesis with heat-cured acrylic material which not only matched the patient’s hand esthetically but was also easy to use. Finger prosthesis offered both esthetic as well as functional rehabilitation to the patient.

## Introduction

The human hand is the most active and extensively used to complete everyday tasks such as holding, eating, writing, and cooking. Traumatic injuries are more commonly associated with amputated fingers. Congenital defects or malformations also need the replacement of missing fingers. They all present a similar clinical challenge during the rehabilitation of amputated fingers [1]. The psychological jolt after the traumatic loss of a finger is even more devastating than the extent of the mutilation [2,3]. Amputation of even the tip of a digit emotionally upsets patients requiring real monitoring [3]. Even the traumatic loss of one finger can cause serious functional as well as psychological impairment.

The highest degree of disability is suffered by individuals whose profession demands the use of fingers. A musician, missing even a part of one finger, will be seriously handicapped. When surgical reconstruction is not possible, prosthetic rehabilitation should be considered. Various types of materials such as polyurethane, polyvinyl chloride, silicone, and acrylic resins can be used for fabricating finger prostheses [4-9]. The prosthetic rehabilitation of such patients should consider the functional, emotional, social, as well as professional factors of the patient.

This case report describes the rehabilitation of a missing distal phalange of the ring finger with a custom-designed acrylic finger prosthesis.

## Case presentation

**Figure 1:**
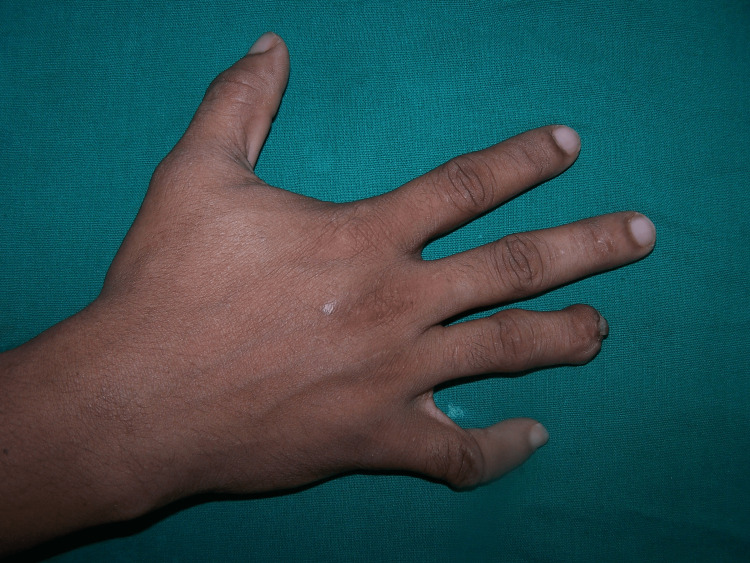
Missing distal phalange of the finger.

A young 26-year-old patient presented for reconstruction of a missing distal phalange of the ring finger in the Department of Prosthodontics (Figure 1).

During history taking, the patient revealed that he had lost his finger in a bike accident. He also revealed that he was a musician by profession and played piano. The sensitive tip of the finger created hindrance while playing piano. Additionally, the patient was concerned about the aesthetics. On examination, a healed scar was seen at the base of the amputated ring finger, brown in color with some crusting. The remaining part of the finger was normal and did not show signs of any infection over the wound. Various types of materials for the fabrication of the finger prosthesis were presented to the patient. Considering the cost, the patient opted for heat-cured acrylic material.

Technique

**Figure 2:**
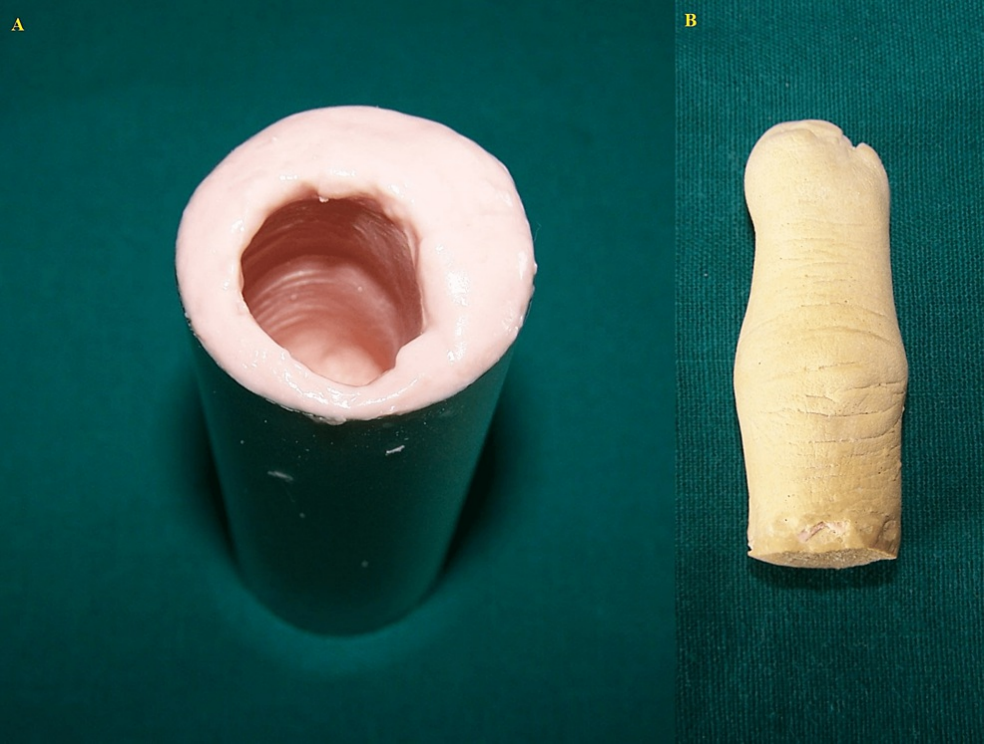
A: impression; B: model.

The patient was treated with a calm and caring attitude. He was asked to keep his hand in a relaxed position. The entire hand was greased with a fine layer of petroleum jelly to allow easy removal of impression material from the finger. An empty plastic cylinder of the same size as the middle finger was used to make an impression. Negative casting was made with alginate (Tropicalgin; DPI, India) by placing over and below the amputated finger. The patient was advised to hold the hand in a normal relaxed position without stretching. The impression was then poured into the dental stone with utmost care to prevent voids (Figure 2).

**Figure 3:**
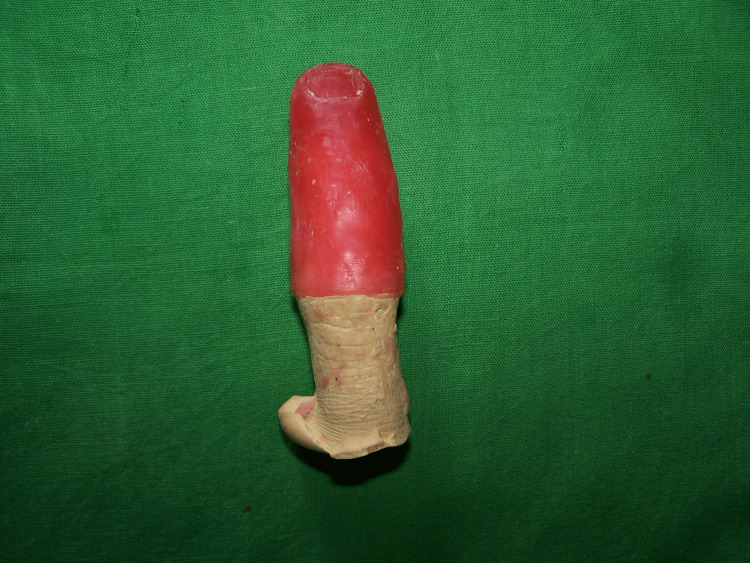
Wax up of the finger.

The remaining finger on the cast which would contact the finger prosthesis was circumferentially scrapped 1 mm uniformly to get a snug fit. On retrieval, the positive model of the finger was sculpted in modeling wax (Figure 3).

**Figure 4:**
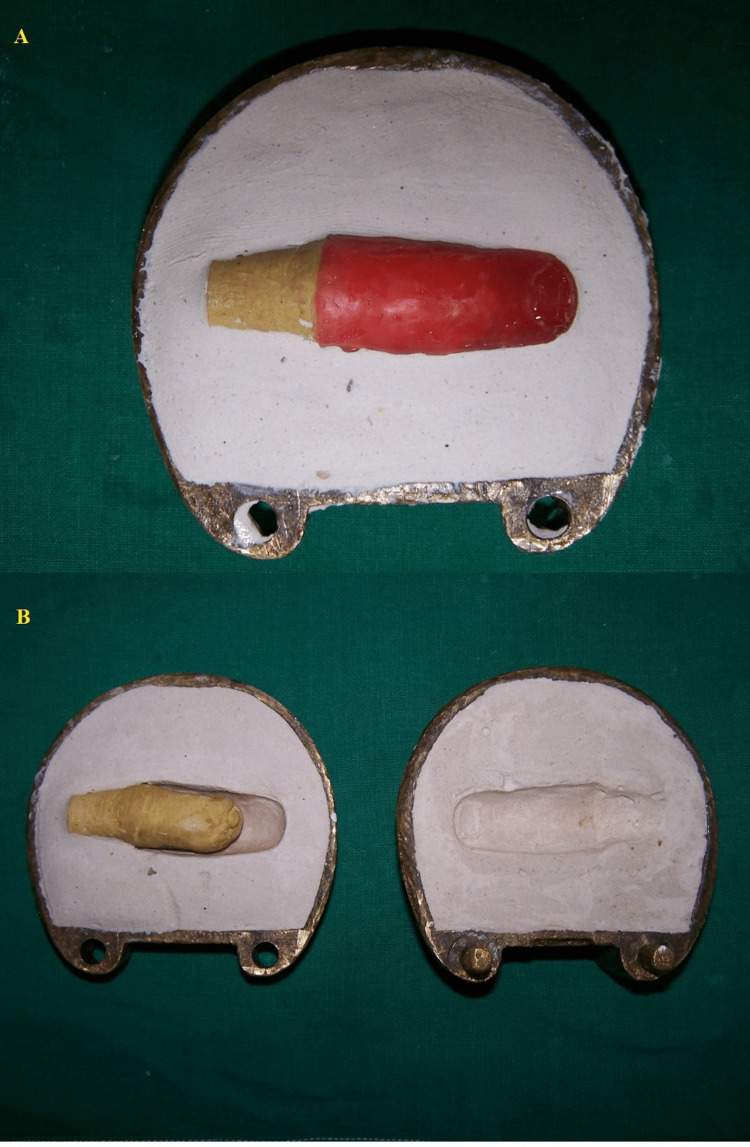
A: investing; B: dewaxing.

The wax finger was placed on the amputated finger to evaluate for correct shape and size with adjoining fingers. After investing and dewaxing, brown color was added in monomer and the clear heat-cured polymer was added to give the prosthesis the look of real skin (Figure 4).

**Figure 5:**
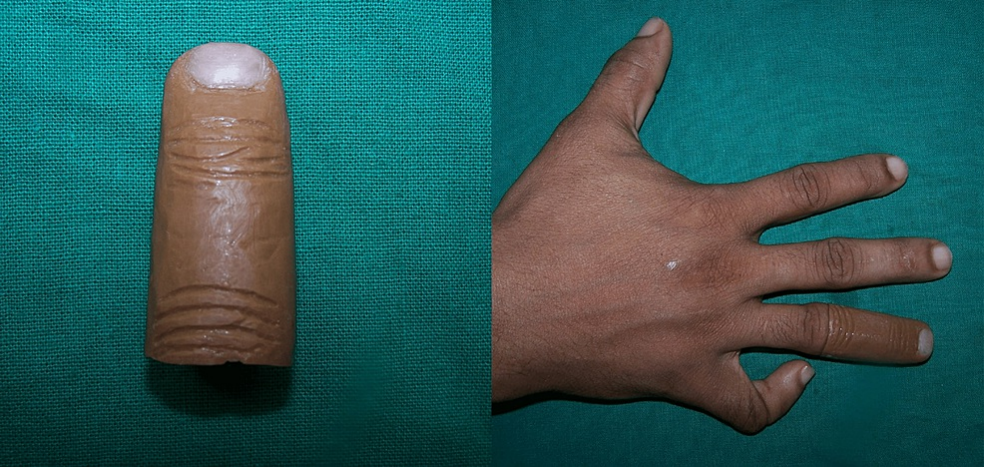
Patient with finger prosthesis.

The finger was processed by heat curing with heat-cured acrylic resin using a conventional technique. On retrieval of the finger prosthesis, the final finishing and polishing were done. Skin creases were created with a bur. An additional external painting was performed to get the exact shade in the finished prosthesis. This was done in front of the patient in natural daylight to gain his approval. To complete the prosthesis the nail portion was made by mixing pink and clear auto-polymerizing resin to match the patient’s adjacent nail. The patient was instructed on how to use and maintain the prosthesis (Figure 5).

The patient was advised to clean the prosthesis inside and outside using soap and lukewarm water. The patient was also advised to keep the prosthesis away from strong solvents to make it last a long time. The patient was recalled frequently to understand his satisfaction related to comfort, fit, and aesthetics. The patient was very happy with the function and aesthetics during the three-month, six-month, and one-year follow-ups.

## Discussion

Fingers play a crucial role in daily tasks, social interaction, as well as aesthetics. Finger amputations are commonly associated with trauma, infection, congenital defects, and infections. Prosthetic rehabilitation is considered when patients cannot afford it or surgical reconstruction is not possible. Custom-designed acrylic finger prosthesis replaces a portion or all of an absent finger. In the present case, the patient was presented with various materials such as acrylic resins, silicones, polyurethane elastomers, and polyvinyl chloride polymers [4-9]. In patients with no financial constraints, three-dimensional-printed fingers can also be a quick and easy alternative. Due to financial constraints, the patient opted for heat-cured acrylic resin material. Reddy et al. successfully used an acrylic finger prosthesis for a partially missing right-hand index finger [10].

Retention of finger prostheses is a prime requisite for proper function and aesthetics. It depends on meticulous planning, proper impression, and scraping of the cast. Retention can also be achieved with the help of implants [5], finger rings [6], medical-grade adhesives [7], and proper scraping of the cast to achieve good contact with the tissues. Leow et al. reported that a 5-7% circumference reduction on the finger model provided a good prosthetic fit for distal finger amputations for thimble-type prostheses [11]. In this case report, a 2 mm uniform circumferential reduction of the stump was carried out to provide a passive vacuum fit.

The finger prosthesis safeguarded the sensitive tip of the finger from trauma and extreme temperatures. It allowed the patient to play piano keys conveniently. It also served as a great psychological benefit to the patient. Making a prosthesis look real with the rest of the hand requires not only creative skill but also specialized expertise.

## Conclusions

Prosthetic rehabilitation offers not only psychological but functional benefits as well. It also allows the surgical site to be easily monitored. Here, we presented the fabrication of a finger prosthesis for an amputated finger. The fabrication of the acrylic finger prosthesis was simple and fast. The prosthesis was fabricated with routinely available materials used for denture fabrication. The patient adapted very well to the prosthesis when recalled frequently.
